# Dietary Protein Requirement of Juvenile *Tor sinensis* Based on Growth Performance, Antioxidants, Digestive Enzyme Activity and Intestinal Morphology

**DOI:** 10.3390/ani16091284

**Published:** 2026-04-22

**Authors:** Yang Yang, Tian Zhong, Huiping Ding, Feng Chen, Yuesong Wang, Rui Cheng, Qi Su, Zhiming Zhang

**Affiliations:** 1 Institute of Hydroecology, Ministry of Water Resources and Chinese Academy of Sciences, Wuhan 430079, China; 2 Hubei Key Laboratory of Animal Nutrition and Feed Science, School of Animal Science and Nutritional Engineering, Wuhan Polytechnic University, Wuhan 430023, China; 3 Innovation Team of the Chang Jiang Water Resources Commission for River and Lake Ecosystem Restoration Key Technology, Wuhan 430079, China

**Keywords:** *Tor sinensis*, dietary protein, growth, antioxidants, digestive enzyme, intestinal morphology

## Abstract

*Tor sinensis* is an emerging aquaculture species in China, yet the effect of dietary protein on its hepatic and intestinal health remains unexplored. This study evaluated the effects of five diets designed to be isoenergetic and isolipidic with graded protein levels (28% to 40%) on juvenile *T. sinensis* (initial weight: 10 ± 0.4 g) over 60 days. Growth performance improved with increasing protein up to 34%, beyond which it plateaued. Regression analysis indicates that the optimal dietary protein level for both weight gain and specific growth rate is 34.3%. Hepatic antioxidant enzyme activities (SOD and CAT) were highest in the 34% protein group, while triglyceride content was lowest. Histological examination revealed minimal hepatocyte swelling, nuclear displacement, and lipid droplet accumulation at this protein level. Intestinal trypsin activity and villus morphology (height, width, and muscular thickness) were also optimized at 34% protein, though lipase and amylase activities remained unaffected. These findings demonstrate that a 34% dietary protein level supports optimal growth, enhances liver antioxidant capacity, and improves intestinal structure and function in juvenile *T. sinensis*, providing critical insights for its formulated feed development.

## Simple Summary

*Tor sinensis*, a fish species with growing potential for aquaculture in China, needs specially formulated feed for healthy and efficient farming. However, little research is available on the nutrient content in the feed specific to this fish. This study aimed to find the best amount of dietary protein used in *T. sinensis* feed. Juvenile *T. sinensis* were fed with five different feeds containing varying protein levels for two months. Results showed that fish grew best when the protein level reached 34% in diet. Moreover, this protein feed enabled the fish not only to grow well but also to develop a healthier liver with enhanced self-protection ability, along with improved gut structure for better nutrient absorption. The results implied that feed containing 34% protein is optimal for the growth and health of juvenile *T. sinensis*. This finding provides clear, science-based guidelines for farmers and supports the development of a sustainable aquaculture industry for this species.

## 1. Introduction

*Tor sinensis* (Cyprinidae) is an economically important cyprinid fish native to the mid-lower reaches of the Lancang (Mekong) River and its tributaries [1]. It is valued both as a palatable food fish, owing to its large size, and in the ornamental trade, due to its bright coloration and culturally auspicious connotations [2]. However, its wild populations are declining, and the species is now classified as vulnerable (VU) on the IUCN Red List [3], primarily due to hydropower development, habitat degradation, overfishing, and pollution [4,5]. Consequently, the development of aquaculture for *T. sinensis* is a pivotal strategy for reconciling its conservation with sustainable utilization.

In aquaculture, feed represents a primary determinant of growth and health. Dietary protein is fundamental, as it supplies essential amino acids for somatic growth, regulates immune and metabolic functions, and influences overall physiological homeostasis. The quantity of dietary protein significantly affects growth performance, metabolic efficiency, and tissue health in farmed fish [6]. Insufficient protein intake limits growth and tissue development [7], while excess protein increases nitrogenous waste, elevates the metabolic burden on the liver, and may disrupt oxidative balance [8]. The optimal dietary protein level is highly species-specific. Therefore, determining this requirement is a critical first step in formulating efficient, cost-effective feeds for *T. sinensis*.

To date, research on *T. sinensis* has focused on areas such as resource assessment [9], phylogenetics [10], habitat modeling [11], artificial propagation [12], and muscle composition [2]. Notably, there is a complete lack of scientific information on its nutritional requirements. No study has investigated the optimal dietary protein level for this species, and the effects of dietary protein on key physiological aspects—such as hepatic antioxidant function and intestinal health—remain entirely unexplored.

Given this critical knowledge gap, the present study was conducted to determine the dietary protein requirement of juvenile *T. sinensis* by evaluating growth performance, antioxidant capacity, digestive enzyme activity and intestinal morphology. The findings of this study are expected to provide the first essential nutritional parameters for formulating a standardized, science-based feed for this vulnerable species. This will serve as a fundamental step towards establishing sustainable and economically viable aquaculture practices for *T. sinensis*, ultimately contributing to the conservation of its wild populations and the responsible development of its commercial potential.

## 2. Materials and Methods

### 2.1. Experimental Diets

To facilitate the examination of the specific effects of protein levels under optimal nutritional conditions, five isonitrogenous and isoenergetic experimental diets were formulated. Since the protein requirement of *T. sinensis* is unknown, protein levels were set at 28%, 31%, 34%, 37%, and 40% (dry matter basis), corresponding to groups P1 to P5, with a narrow 3% increment to facilitate accurate breakpoint estimation via polynomial regression [13].

Fish meal, casein, and soybean meal served as protein sources, while fish oil and soybean oil were the primary lipid sources. To maintain isoenergetic and isolipidic conditions across diets, the inclusion levels of the lipid sources were held constant. Adjustments in protein content were achieved by modifying the proportions of the protein ingredients. Corresponding changes in digestible energy and diet bulk were compensated by inversely varying the inclusion of a non-protein energy substrate (wheat flour). The proximate composition (crude protein, lipid, and gross energy) of each diet was calculated based on the analyzed nutrient composition of individual ingredients (data provided by suppliers) and was subsequently verified by chemical analysis (Table 1).

All dry ingredients were ground and passed through a 60-mesh sieve. The powdered ingredients were thoroughly mixed, after which atomized oil and water were gradually added to form a uniform dough. The mixture was then processed using a twin-screw extruder (Jinan Saixin Mechanical Equipment Co., Ltd., Jinan, China) to produce pellets with a diameter of 2 mm and a length of approximately 1 mm. The pellets were air-dried in a cool, dry environment and stored at −20 °C until use.

### 2.2. Experimental Fish and Feeding Management

The experiment was conducted at the Nuozhadu Fish Breeding Station in Puer City, Yunnan Province. Juvenile *T. sinensis* used in this trial were the offspring (F1 generation) of wild adults captured from the Lancang River. A total of 1000 six-month-old fish were acclimatized to laboratory conditions for two weeks. Subsequently, 450 juveniles (average body weight: 10.00 ± 0.40 g) were randomly distributed into 15 cylindrical fiberglass-reinforced plastic (FRP) tanks (1 m in diameter × 0.8 m depth) in a recirculating aquaculture system. The tanks were allocated to five dietary treatment groups, with each group comprising three replicate tanks (30 fish per tank).

The feeding trial lasted for 60 days. Fish were fed one of the experimental diets to apparent satiation twice daily (08:00 and 18:00). Thirty minutes before each feeding, water circulation and aeration were temporarily halted to allow for the siphoning removal of feces and any accumulated waste. The weight of feed offered at each meal was recorded. Uneaten feed was collected by siphoning 30 min post-feeding, oven-dried, and weighed to determine feed consumption. Throughout the experimental period, the water depth was maintained at approximately 0.6 m. Water quality parameters were maintained within the following ranges: temperature, 26–28 °C; dissolved oxygen, 7.3–7.8 mg·L^−1^ and pH, 7.3–7.5; total ammonia nitrogen (TAN), <0.5 mg·L^−1^; nitrite nitrogen (NO_2_^−^-N), <0.1 mg·L^−1^.

### 2.3. Sample Collection

At the end of the feeding trial, all fish were fasted for 24 h. Subsequently, five fish per tank were randomly selected, anesthetized with MS-222 (20 mg·L^−1^, Sigma-Aldrich, St. Louis, MO, USA), and measured for total length, body length, and body weight (to the nearest 0.01 cm and 0.01 g, respectively).

The anesthetized fish were then dissected. Livers were immediately excised. From each fish, a portion of the liver was snap-frozen in liquid nitrogen and stored at −80 °C for subsequent enzyme activity analysis. Another portion was fixed in 4% paraformaldehyde for 24 h at 4 °C for subsequent paraffin embedding, sectioning, and histological examination. Intestines were carefully removed, and adherent adipose tissue was cleared. Approximately 1 cm segments from the anterior, middle, and posterior regions (representing the foregut, midgut, and hindgut) were collected and fixed in 4% paraformaldehyde for histological evaluation. A segment of the midgut was collected from each fish, snap-frozen in liquid nitrogen, and stored at −80 °C for subsequent analysis of digestive enzyme activities.

### 2.4. Analysis of Diet Composition

The proximate composition of the experimental diets was determined using standard methods of the Association of Official Analytical Chemists (AOAC) [14]. Moisture content was determined by oven-drying at 105 °C to constant weight. Crude protein (N × 6.25) was analyzed using the Kjeldahl method with a Kjeltec 8400 Analyzer (FOSS, Hillerød, Denmark). Crude fat was extracted with diethyl ether using a Soxhlet apparatus (FOSS, Hillerød, Denmark). Crude ash content was determined by combustion in a muffle furnace at 550 °C for 6 h. Gross energy was measured using an isoperibol oxygen bomb calorimeter (HY-9M, Huayue Instrument Co., Shenzhen, China) following the standard procedure described by FAO [15]. All analyses were performed in triplicate, and results are expressed on a dry matter basis.

### 2.5. Growth Performance Indicators and Calculation Formulas

Growth conditions of fish were evaluated using weight gain rate (WGR), specific growth rate (SGR), feeding ratio (FR), feed conversion rate (FCR), survival rate (SR), and condition factor (CF); each indicator was calculated as follows:

WGR = 100 × (Wt − W0)/W0;

SGR = (ln Wt − ln W0) × 100/t;

FR = 100 × F/[t × (Wt + W0)/2];

FCR = F/(Wt − W0);

SR = 100 × (Nt − N0)/N0;

CF = 100 × Wt/L^3^.

where W0 is the initial weight (g); Wt is the final weight (g); t is the number of days of the experiment; L is the standard length (cm); N0 is the initial number of fish; Nt is the final number of fish; and F is the diet (g) [16].

### 2.6. Measurement of Liver and Intestinal Biochemical Indices

Hepatic and intestinal enzyme activities (or content) were determined using commercial assay kits (Nanjing Jiancheng Bioengineering Institute, Nanjing, China). The specific kits and their catalog numbers were as follows: superoxide dismutase (SOD, A001-1), catalase (CAT, A007-1-1), triglyceride (TG, A110-1-1), total antioxidant capacity (T-AOC, A015-2-1), and lysozyme (LZM, A050-1-1) for liver tissue; trypsin (TPS, A080-2), lipase (LPS, A054-1-1), and α-amylase (α-AMS, C016-1-1) for intestinal tissue.

Tissue samples (approximately 0.1 g) were homogenized in 0.9 mL of ice-cold normal saline (0.9% NaCl) and centrifuged at 3000× *g* for 10 min at 4 °C. The resulting supernatant was collected for analysis. All assays were conducted according to the manufacturer’s protocols, and absorbance was measured using a microplate reader (BioTek Synergy H1, Winooski, VT, USA).

The specific methodologies were as follows: SOD activity was determined using the WST-1 method, based on the inhibition of superoxide anion-mediated formazan formation. CAT activity was measured by monitoring the decomposition of H_2_O_2_ at 240 nm. TG content was determined enzymatically using glycerol-3-phosphate oxidase. T-AOC was assessed via the ferric reducing antioxidant power (FRAP) assay. LZM activity was quantified by measuring the lysis rate of *Micrococcus lysodeikticus*. For intestinal enzymes, TPS activity was assayed using casein as a substrate (absorbance at 275 nm), LPS activity was determined titrimetrically with an olive oil emulsion, and α-AMS activity was measured using the 3,5-dinitrosalicylic acid (DNS) method to quantify maltose release from starch. Three replicate tanks were used per dietary group (experimental unit, *n* = 3). Five fish were sampled from each tank, and data were averaged at the tank level prior to statistical analysis.

### 2.7. Liver and Intestinal Histological Indices

Liver and intestinal segments fixed in 4% paraformaldehyde were processed using standard histological techniques. Tissues were dehydrated through a graded ethanol series, cleared in xylene, embedded in paraffin, and sectioned at 5 μm thickness. After deparaffinization and rehydration, sections were stained with hematoxylin and eosin (H&E) for general morphological evaluation.

Hepatic lipid deposition was assessed by Oil Red O staining of neutral lipids. Frozen liver tissues were sectioned at 8 μm thickness in a cryostat, fixed in 10% neutral buffered formalin for 10 min, rinsed, and stained with a filtered Oil Red O working solution. Nuclei were counterstained with hematoxylin.

All stained sections were observed and imaged under a light microscope (CX33, SOPTOP Sunny, Ningbo, China) equipped with a digital camera. For the intestine, villus height (VH), villus width (VW), and muscularis thickness (MT) were measured from at least 10 well-oriented villi per fish using image analysis software (ImageJ 1.52a, NIH, Bethesda, MD, USA). Liver histology was evaluated qualitatively for structural changes.

### 2.8. Statistical Analysis

The tank was considered the experimental unit for all growth, biochemical, and histological analyses. Data from five individual fish within each tank were averaged to obtain a single value per tank, and the tank was used as the independent experimental unit (*n* = 3 per dietary treatment) to avoid pseudoreplication. Differences in mean values between groups were analyzed using SPSS 28.0 software. After testing for normality and homogeneity of variance, polynomial contrasts were performed to evaluate linear and quadratic responses. All data were subjected to one-way analysis of variance (ANOVA) followed by Tukey’s Honestly Significant Difference (HSD) test. A significance level of *p* < 0.05 was considered statistically significant. Results are expressed as mean ± standard deviation (mean ± SD).

## 3. Results

### 3.1. Effects of Dietary Protein Levels on the Growth Performance of Juvenile T. sinensis

**Figure 1:**
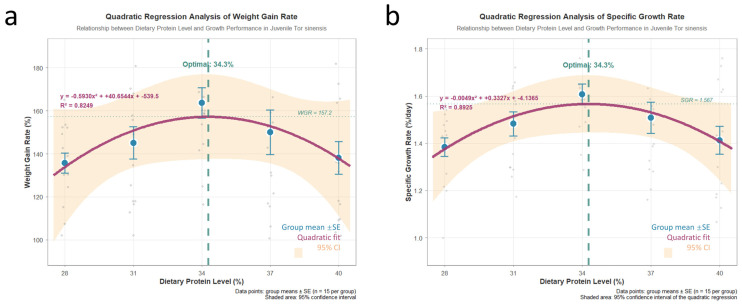
Physiological parameters of juvenile *T. sinensis* fed with various protein levels ((**a**): weight gain rate; (**b**): specific growth rate).

Dietary protein level significantly influenced the growth performance of juvenile *T. sinensis* (Table 2). Growth indicators, including weight gain rate (WGR), and specific growth rate (SGR), increased with increasing dietary protein levels, peaking in the P3 group, which showed significantly higher values than all other groups (*p* < 0.05). Conversely, the feed conversion ratio (FCR) was significantly lower in the P3 group compared to other groups (*p* < 0.05). No significant differences were observed in survival rate among treatments (*p* > 0.05). A second-order polynomial regression analysis based on WGR/SGR data estimated the optimal dietary protein level to be 34.3% (Figure 1).

### 3.2. Effects of Dietary Protein Levels on Liver Biochemical Indices in Juvenile T. sinensis

As shown in Table 3, the activities of hepatic superoxide dismutase (SOD) and catalase (CAT) increased with increasing dietary protein levels, peaked in the P3 group, and then gradually declined. The values in the P3 group were significantly higher than those in the other groups (*p* < 0.05). Conversely, hepatic triglyceride (TG) content first decreased and then increased with higher dietary protein levels, with the P3 group showing a significantly lower TG content than the other groups (*p* < 0.05). In addition, no significant differences (*p* > 0.05) were observed among groups for the level of total antioxidant capacity (T-AOC) or the activity of lysozyme (LZM) in the liver.

### 3.3. Effects of Dietary Protein Levels on Liver Morphology in Juvenile T. sinensis

**Figure 2:**
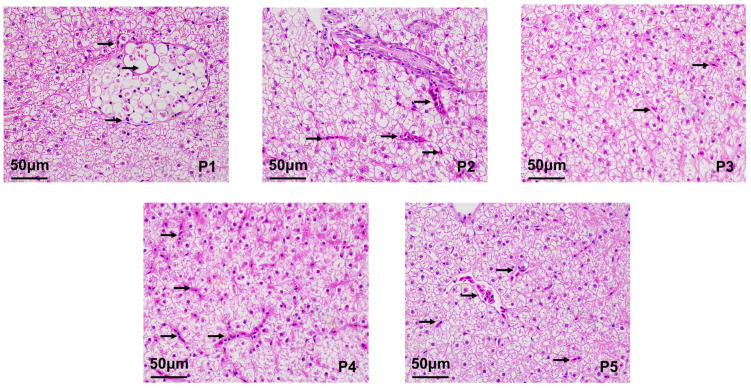
Effects of different protein levels on the liver (HE-stained sections). Note: Scale bar = 50 μm. The arrows indicate hepatocyte swelling, nuclear displacement, and cellular vacuolation.

Although all the groups presented relatively intact, clear boundaries between cells and no signs of inflammatory cell infiltration of the liver (Figure 2), hepatocyte swelling, nuclear displacement, and vacuolization were observed. The P1 group exhibited pronounced vacuolization of liver cells, while the P2, P4 and P5 groups showed more frequent nuclear displacement. In contrast, the P3 group had significantly fewer instances of cell swelling and nuclear displacement compared to the other groups.

**Figure 3:**
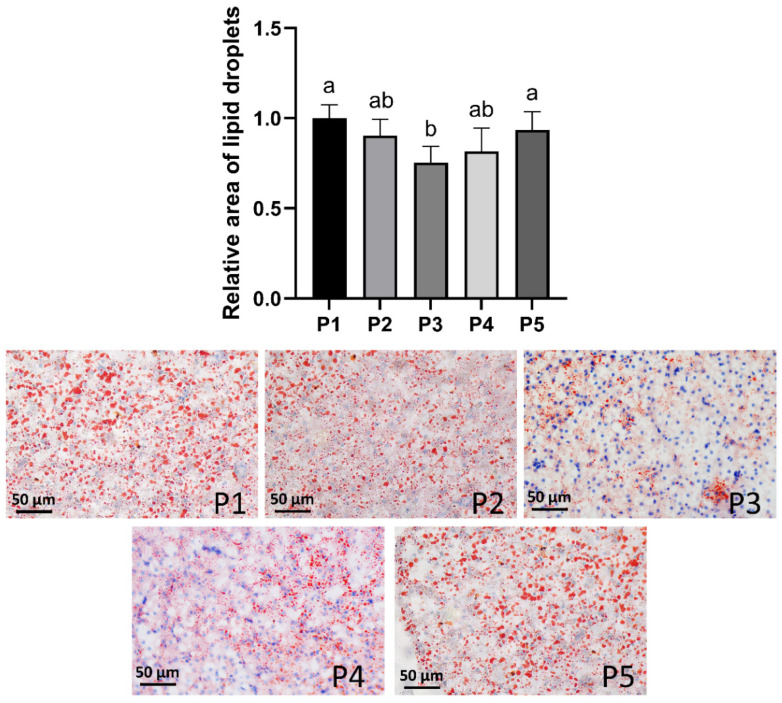
Effects of different protein levels on the liver (Oil Red O staining) and relative area of lipid droplets. Data are presented as mean ± standard error (SE). Different lowercase letters indicate significant differences among groups (*p* < 0.05). Note: Scale bar = 50 μm.

In Figure 3, hepatic lipid deposition was evaluated using Oil Red O staining. The results of liver lipidosis staining were quantified using ImageJ. Juvenile fish in the P1 group exhibited abundant and enlarged lipid droplets, indicating pronounced hepatic lipid accumulation under the lowest dietary protein level. As dietary protein increased from 28% to 34%, both the number and size of lipid droplets showed a progressive reduction. Notably, the P3 group presented the minimal lipid deposition among all treatments, characterized by markedly fewer and smaller lipid droplets. However, further increases in dietary protein (P4 and P5) resulted in a renewed elevation in hepatic lipid droplet abundance and size. Overall, the pattern demonstrated a protein-dependent biphasic response, with the lowest hepatic lipid accumulation observed at 34% dietary protein.

### 3.4. Effects of Dietary Protein Levels on Intestinal Biochemical Indices in Juvenile T. sinensis

As shown in Table 4, dietary protein level significantly influenced intestinal trypsin activity. The activity increased with increasing protein levels, peaking in the P3 group, and then decreased at higher levels. In contrast, the activities of lipase and amylase were not significantly affected by dietary protein levels (*p* > 0.05).

### 3.5. Effects of Dietary Protein Levels on Intestinal Morphology in Juvenile T. sinensis

**Figure 4:**
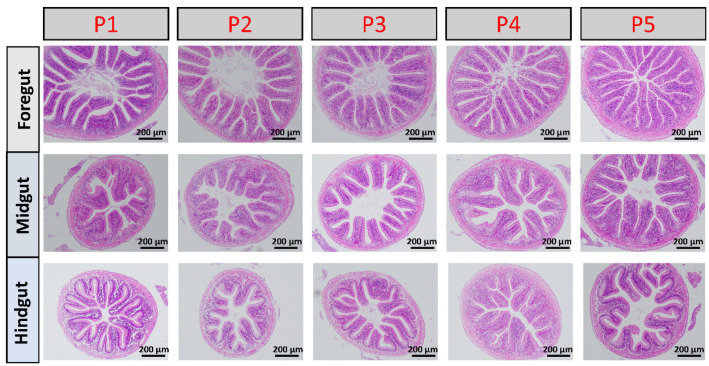
Effects of different protein levels on intestinal tissue morphology (HE staining). Note: Scale bar = 200 μm.

The morphology in intestine of juvenile *T. sinensis* fed experimental diets was examined by H&E. As shown in Table 5 and Figure 4, VH, VW, and MT in the foregut increased and then decreased with increasing dietary protein levels. VH in the foregut of the P3 group was significantly higher than those of other groups (*p* < 0.05), while the remaining indicators showed no significant differences (*p* > 0.05). Dietary protein levels had no significant effects on VH or MT in the midgut (*p* > 0.05), but VW in the P3 group was significantly greater than those in other groups (*p* < 0.05). In the hindgut, both VW and MT increased, reaching the highest level at P3 group, and then decreased with increasing dietary protein levels, whereas VH showed no significant difference among all groups (*p* > 0.05).

## 4. Discussion

### 4.1. Effects of Dietary Protein Levels on Growth Performance

Dietary protein is the primary source of essential amino acids for fish growth and tissue accretion, and its level directly determines the balance between anabolic demand and metabolic cost [17]. In the present study, the growth performance of juvenile *T. sinensis* exhibited a clear quadratic response to increasing dietary protein levels, with final body weight, WGR, SGR, and CF all peaking at approximately 34% protein. This response pattern indicates that both protein deficiency and excess can constrain growth efficiency.

At the low protein level (28%), insufficient availability of indispensable amino acids likely limited muscle protein synthesis and cellular proliferation, thereby restricting somatic growth [18]. In contrast, although higher protein diets (37–40%) supplied excess amino acids, growth performance did not further improve, and feed conversion efficiency declined. This suggests that surplus dietary protein exceeded the physiological capacity for efficient utilization and was increasingly catabolized for energy, leading to elevated nitrogen excretion and metabolic burden [8]. Similar patterns of reduced protein utilization efficiency at excessive dietary protein levels have been reported in juvenile grass carp and common carp [19,20].

The lowest FCR observed at 34% protein further supports the conclusion that this level represents an optimal balance between nutrient supply and utilization efficiency. Notably, the FCR values obtained in this study fall within the typical range reported for juvenile omnivorous cyprinids under controlled feeding conditions [21,22]. Together, these findings indicate that approximately 34% dietary protein most effectively supports somatic growth of juvenile *T. sinensis* by maximizing protein retention while minimizing metabolic inefficiency.

### 4.2. Effects of Dietary Protein Levels on Liver Antioxidant Capacity and Morphology

The liver plays an indispensable role in nutrient metabolism, detoxification, and energy regulation in fish [23]. As the central organ responsible for oxidative metabolism, the liver maintains physiological homeostasis through antioxidant enzymes that neutralize excessive reactive oxygen species (ROS) generated under nutritional or environmental stress [24,25]. Enzymes such as SOD and CAT are essential for maintaining redox balance, protecting hepatocytes from oxidative injury, and ensuring normal growth and development [26,27]. In this study, hepatic antioxidant enzyme activities (SOD and CAT) increased with dietary protein level up to 34% and declined thereafter, suggesting that an optimal protein supply enhances hepatic redox homeostasis, whereas both deficiency and excess disrupt antioxidant balance [28].

At suboptimal protein levels, limited amino acid availability may restrict the synthesis of antioxidant enzymes and redox-related proteins, thereby weakening the liver’s capacity to neutralize reactive oxygen species (ROS) [24]. Conversely, excessive dietary protein can intensify amino acid deamination and mitochondrial oxidative metabolism, leading to elevated ROS production and oxidative stress [29]. This dual effect provides a mechanistic explanation for the observed decline in SOD and CAT activities at higher protein levels.

Hepatic triglyceride content reached its lowest level at 34% protein, indicating improved lipid utilization under optimal protein intake. Similar reductions in hepatic lipid accumulation at appropriate dietary protein levels have been reported in other cyprinid species [30]. Histological observations further supported these biochemical results: pronounced hepatocyte vacuolization and nuclear displacement were evident in fish fed low- and high-protein diets, whereas fish in the 34% protein group exhibited improved cellular integrity. Oil Red O staining confirmed a protein-dependent biphasic response in hepatic lipid deposition, with minimal lipid accumulation at the optimal protein level.

In contrast, total antioxidant capacity (T-AOC) and lysozyme (LZM) activity showed no significant differences among groups (*p* > 0.05), despite observable numerical trends. The lack of significance, coupled with the relatively high standard deviations for these parameters (Table 3), may reflect greater individual variation in these integrated, non-enzymatic antioxidant pools and immune markers compared to specific enzyme activities. It could also indicate that the dietary protein range tested was not a primary driver of these broader systemic parameters under the present experimental conditions.

Collectively, these findings indicate that an appropriate dietary protein level supports hepatic antioxidant defenses and lipid metabolic homeostasis, while deviations from this optimum—either insufficient or excessive protein—can impose oxidative and metabolic stress on the liver.

### 4.3. Effects of Dietary Protein Levels on Intestinal Digestive Enzymes and Morphology

As an omnivorous fish, *T. sinensis* relies heavily on intestinal digestion and absorption for nutrient utilization [31]. The intestine is a key organ responsible for digestion, absorption, immunity, and endocrine regulation. Therefore, the structural integrity of intestinal villi and muscular layers directly affects nutrient uptake efficiency, digestive enzyme activity and overall health status in fish [32]. In the present study, intestinal trypsin activity increased with dietary protein level and peaked at 34%, indicating enhanced proteolytic capacity under optimal protein supply. Similar protein-dependent responses of intestinal protease activity have been reported in *Erythroculter ilishaeformis*, *Pelteobagrus vachelli*, and *Barbodes caldwelli* [33,34,35].

Excessive dietary protein, however, did not further enhance trypsin activity and instead resulted in a declining trend, suggesting that overly high protein intake may impair digestive efficiency through metabolic overload or feedback inhibition, as observed in other cyprinids and omnivorous fishes [36,37]. In contrast, lipase and α-amylase activities were not significantly affected by dietary protein levels, consistent with findings in crucian carp and other omnivorous fish species [38,39,40]. This may reflect the specific nutritional priorities and metabolic partitioning in *T. sinensis*. Firstly, dietary protein is primarily directed towards growth and somatic protein synthesis rather than lipogenesis or glucogenesis. Therefore, the activities of fat- and carbohydrate-digesting enzymes may be regulated more by their respective dietary substrate levels than by protein intake [41]. Secondly, the experimental diets were isoenergetic and isolipidic; the constant lipid supply likely provided a stable substrate signal for lipase, preventing significant induction or repression by varying protein levels. Similarly, the non-protein energy fraction remained relatively constant across diets, which could explain the stable α-amylase activity. This functional dissociation suggests that the digestive system of *T. sinensis* modulates protease activity adaptively in response to dietary protein, while maintaining basal capacities for lipid and carbohydrate digestion, a strategy that may optimize metabolic efficiency under varying nutrient availabilities.

Intestinal histology analysis revealed that villus height, villus width, and muscular thickness were generally enhanced at the optimal protein level, particularly in the foregut and hindgut. Increased villus dimensions expand the absorptive surface area and improve nutrient uptake efficiency, while enhanced muscular thickness supports intestinal motility [42]. The concordance between elevated trypsin activity and improved intestinal morphology at 34% protein suggests that optimal dietary protein intake enhances both functional and structural aspects of intestinal digestion. The considerable individual variation in these morphological measurements, reflected in the standard deviations, likely contributed to the lack of statistical power. This variation is common in histological studies and can arise from slight differences in sectioning angle, plane of measurement, and inherent biological variability between fish.

### 4.4. Mechanistic Interpretation and Biological Implications

The integrated multi-organ analysis in this study reveals that a dietary protein level of approximately 34% establishes a physiological optimum for juvenile *T. sinensis* by synchronizing anabolic processes with metabolic and oxidative homeostasis. We propose that this synchrony is mediated through protein-sensing and nutrient-signaling pathways that coordinate responses across tissues.

The optimal protein supply likely supports efficient growth by providing both sufficient essential amino acids as substrates and the necessary amino acid signaling (via the mTOR pathway) to activate hepatic and muscular protein synthesis [43,44]. Concurrently, adequate amino acid availability may enhance the synthesis of antioxidant enzymes (SOD and CAT) themselves, which are proteins, and support the hepatic transsulfuration pathway for glutathione synthesis, thereby bolstering the antioxidant defense system [43].

The observed reduction in hepatic lipid deposition (TG content) at the optimal protein level suggests a shift in energy metabolism from lipid storage towards protein-directed anabolism. This could be facilitated by the role of certain amino acids in modulating lipid catabolism and inhibiting de novo lipogenesis in the liver [45]. In the intestine, the upregulation of trypsin activity alongside improved villus architecture at 34% protein indicates a nutrient-mediated adaptive response, where optimal dietary substrate levels enhance both the functional capacity and absorptive surface of the digestive organ, possibly through local growth factor signaling (IGF) [46].

Conversely, suboptimal or excessive protein disrupts this coordinated network. Deficiency starves these pathways, limiting their output. Excess protein, however, imposes a metabolic overload: increased amino acid catabolism in the liver may elevate ammonia production and reactive oxygen species generation, explaining the decline in antioxidant enzyme activities and the rise in oxidative stress markers at higher protein levels [47]. The energy cost of deaminating and excreting surplus nitrogen may also divert energy from growth and compromise overall metabolic efficiency.

Therefore, the dietary protein requirement for *T. sinensis* should be defined not merely as the level for maximum growth but as the level that sustains a harmonious equilibrium across anabolic, antioxidant, and digestive systems. Our findings provide a physiological basis for feed formulation, emphasizing that balanced protein nutrition is key to achieving sustainable aquaculture for this species by optimizing health and efficiency simultaneously.

### 4.5. Limitations and Future Perspectives

Although this study provides the first evidence of the dietary protein requirement of *T. sinensis,* several limitations remain. The current analysis focuses on phenotypic, enzymatic, and histological responses, while molecular regulatory mechanisms—such as the expression of antioxidant genes (*SOD*, *CAT*, and *GPX*) and metabolic pathways—were not investigated. Future studies should integrate transcriptomic, proteomic, or metabolomic approaches to elucidate underlying molecular mechanisms. In addition, long-term feeding trials are recommended to assess whether the identified optimal protein level remains effective across different developmental stages and environmental conditions.

## 5. Conclusions

In conclusion, the present study demonstrates that a dietary protein level of approximately 34% is optimal for juvenile *T. sinensis*. This level optimally supported growth performance, enhanced hepatic antioxidant capacity, improved intestinal digestive function, and maintained healthy tissue morphology. The findings provide essential baseline data for formulating cost-effective and physiologically appropriate feeds for the aquaculture of this species. Further studies are warranted to validate these requirements under different conditions and to elucidate the underlying metabolic mechanisms.

## Figures and Tables

**Table 1:** Formulation and nutrient levels of the experimental diets (dry matter basis).

Ingredients	Groups				
	P1	P2	P3	P4	P5
Protein sources					
Fish meal	20.00	20.00	20.00	20.00	20.00
Soybean meal	25.00	25.00	25.00	25.00	25.00
Casein	3.55	7.25	10.85	14.45	18.25
Other ingredients					
Wheat flour	31.90	28.20	24.60	21.00	17.20
Soybean oil	3.50	3.50	3.50	3.50	3.50
Fish oil	3.50	3.50	3.50	3.50	3.50
Cellulose	8.00	8.00	8.00	8.00	8.00
Ca (H_2_PO_4_)_2_	2.00	2.00	2.00	2.00	2.00
Choline chloride	0.50	0.50	0.50	0.50	0.50
Multivitamin ^1^	1.00	1.00	1.00	1.00	1.00
Mineral mix ^2^	1.00	1.00	1.00	1.00	1.00
Antioxidants ^3^	0.05	0.05	0.05	0.05	0.05
Nutrient levels (measured values)					
Crude protein (g·kg^−1^)	28.25 ± 0.18 ^e^	31.23 ± 0.22 ^d^	34.09 ± 0.21 ^c^	36.95 ± 0.31 ^b^	39.90 ± 0.25 ^a^
Crude fat (g·kg^−1^)	10.59 ± 0.11	10.52 ± 0.15	10.43 ± 0.22	10.50 ± 0.21	10.37 ± 0.19
Crude ash (g·kg^−1^)	8.56 ± 0.12	8.77 ± 0.33	8.56 ± 0.31	8.43 ± 0.25	8.80 ± 0.38
Moisture (g·kg^−1^)	6.89 ± 0.10	7.00 ± 0.16	6.91 ± 0.18	6.96 ± 0.27	7.03 ± 0.32
Crude fiber (%)	10.38 ± 0.25	10.30 ± 0.18	10.25 ± 0.19	10.15 ± 0.30	10.06 ± 0.36
Gross energy (kJ·g^−1^)	14.82 ± 0.17	14.77 ± 0.28	14.81 ± 0.25	14.91 ± 0.13	14.89 ± 0.26

Note: ^1^ Multivitamin per kilogram: VA 10 mg, VB_1_ 80 mg, VB_2_ 100 mg, VB_6_ 60 mg, VB_12_ 0.6 mg, VC 1100 mg, VD_3_ 3.3 mg, VE 400 mg, VK_3_ 90 mg, D-calcium pantothenate 310 mg, niacinamide 600 mg, folic acid 70 mg, inositol 700 mg, D-biotin 1 mg, wheat middling 6.49 g. ^2^ Mineral mix per kilogram: NaF 2 mg, KI 10 mg, CoCl_2_·6H_2_O (1%) 50 mg, CuSO_4_·5H_2_O 76 mg, FeSO_4_·H_2_O 360 mg, ZuSO_4_·H_2_O 170 mg, MuSO_4_·H_2_O 104 mg, MgSO_4_·H_2_O 600 mg, Ca(H_2_PO_4_)_2_·H_2_O 2500 mg, NaCl 100 mg, zeolite powder 6.05 g. ^3^ Antioxidants: Ethoxyquin 15%, Butylated hydroxyanisole 0.2%, Butylated hydroxytoluene 6%, Citric acid 4%, Carriers (silica and zeolite powder). All proximate composition analyses (crude protein, crude lipid, ash, moisture and crude fiber) were performed in triplicate according to AOAC methods [14]. Values are expressed as mean ± standard deviation (SD). In the same row, values with no letter or the same letter superscripts mean no significant difference (*p* > 0.05), while those with different small letter superscripts mean significant difference (*p* < 0.05). The variation among replicates was minimal (CV < 2%). Following a one-way analysis of variance (ANOVA), no statistically significant differences (*p* > 0.05) were observed among all experimental diets for crude fat and total energy content.

**Table 2:** Growth index of juvenile *T. sinensis* under different protein levels.

Index	Groups				
	P1	P2	P3	P4	P5
TL (cm)	13.54 ± 1.03 ^b^	14.01 ± 0.86 ^ab^	14.25 ± 0.77 ^a^	14.07 ± 0.85 ^ab^	13.85 ± 1.09 ^b^
BL (cm)	10.38 ± 0.92	10.55 ± 0.92	10.53 ± 0.71	10.46 ± 0.70	10.24 ± 0.69
FW(g)	9.98 ± 0.38	9.94 ± 0.34	10.07 ± 0.33	10.13 ± 0.27	10.05 ± 0.35
FBW (g)	23.04 ± 2.00 ^b^	24.72 ± 2.92 ^ab^	26.37 ± 2.74 ^a^	25.01 ± 3.41 ^ab^	23.55 ± 3.24 ^b^
WGR (%)	130.70 ± 17.93 ^b^	145.06 ± 19.22 ^ab^	163.65 ± 17.39 ^a^	150.05 ± 20.23 ^ab^	138.15 ± 29.30 ^b^
SGR (%·d^−1^)	1.38 ± 0.15 ^b^	1.48± 0.20 ^ab^	1.61 ± 0.17 ^a^	1.51 ± 0.26 ^ab^	1.41 ± 0.23 ^b^
CF (g·cm^−3^)	2.06 ± 0.22 ^b^	2.10 ± 0.16 ^ab^	2.26 ± 0.18 ^a^	2.20 ± 0.15 ^ab^	2.09 ± 0.2 ^b^
FR (%)	2.43 ± 0.09 ^a^	2.33 ± 0.20 ^ab^	2.21 ± 0.17 ^b^	2.31 ± 0.25 ^ab^	2.41 ± 0.23 ^a^
FCR	1.89 ± 0.36 ^a^	1.72 ± 0.35 ^ab^	1.51 ± 0.26 ^b^	1.70 ± 0.41 ^ab^	1.87 ± 0.45 ^a^
SR (%)	97.78 ± 1.95	95.56 ± 1.11	97.78 ± 1.11	97.78 ± 1.11	95.56 ± 2.22

Note: Values are presented as mean ± SD (*n* = 3 tanks per treatment). For each tank, measurements were obtained from five randomly sampled fish, and the average value per tank was used for statistical analysis. The tank was considered the experimental unit. One-way analysis of variance was used to test the data for significance. In the same row, values with no letter or the same letter superscripts mean no significant difference (*p* > 0.05), while those with different small letter superscripts mean significant difference (*p* < 0.05).

**Table 3:** Liver biochemical indices of juvenile *T. sinensis* under different protein levels.

Index	Groups				
	P1	P2	P3	P4	P5
SOD(U·mL^−1^)	2.02 ± 0.76 ^bc^	1.98 ± 0.06 ^bc^	3.03 ± 0.16 ^a^	2.34 ± 0.20 ^ab^	1.29 ± 0.52 ^c^
CAT (U·mL^−1^)	17.64 ± 7.84 ^c^	24.89 ± 2.54 ^bc^	40.37 ± 2.85 ^a^	31.88 ± 5.03 ^ab^	22.25 ± 10.75 ^bc^
TG (mmol·gprot^−1^)	0.63 ± 0.05 ^a^	0.50 ± 0.21 ^ab^	0.13 ± 0.04 ^c^	0.30 ± 0.17 ^bc^	0.48 ± 0.14 ^b^
T-AOC (mM)	0.77 ± 0.32	0.84 ± 0.27	1.02 ± 0.02	1.00 ± 0.01	0.98 ± 0.03
LZM (U·mL^−1^)	11.05 ± 1.74	11.81 ± 3.43	15.73 ± 16.09	7.30 ± 3.51	5.73 ± 3.31

Note: Values are presented as mean ± SD (*n* = 3 tanks per treatment). For each tank, measurements were obtained from five randomly sampled fish, and the average value per tank was used for statistical analysis. One-way analysis of variance was used to test the data for significance. In the same row, values with no letter or the same letter superscripts mean no significant difference (*p* > 0.05), while those with different small letter superscripts mean significant difference (*p* < 0.05).

**Table 4:** Intestinal biochemical indices of juvenile *T. sinensis* under different protein levels.

Index	Groups				
	P1	P2	P3	P4	P5
TPS(U·mgprot^−1^)	238.52 ± 32.00 ^ab^	288.45 ± 18.67 ^ab^	307.56 ± 106.26 ^a^	303.26 ± 34.41 ^ab^	168.95 ± 101.04 ^b^
LPS(U·gprot^−1^)	27.28 ± 40.97	17.14 ± 7.14	28.35 ± 28.74	15.42 ± 9.39	18.32 ± 8.32
α-AMS(U·mgprot^−1^)	0.85 ± 0.11	0.88 ± 0.14	1.27 ± 0.25	0.79 ± 0.05	0.77 ± 0.04

Note: Values are presented as mean ± SD (*n* = 3 tanks per treatment). For each tank, measurements were obtained from five randomly sampled fish, and the average value per tank was used for statistical analysis. One-way analysis of variance was used to test the data for significance. In the same row, values with no letter or the same letter superscripts mean no significant difference (*p* > 0.05), while those with different small letter superscripts mean significant difference (*p* < 0.05).

**Table 5:** Intestinal villus characteristics of juvenile *T. sinensis* under different protein levels (μm).

Index		Groups				
		P1	P2	P3	P4	P5
Foregut	VH	369.48 ± 115.90 ^b^	396.88 ± 48.68 ^ab^	479.81 ± 50.43 ^a^	453.30 ± 86.52 ^ab^	410.93 ± 80.20 ^ab^
	VW	100.68 ± 9.78	102.25 ± 16.75	111.95 ± 15.99	109.37 ± 30.79	101.87 ± 17.75
	MT	65.33 ± 13.00	75.53 ± 27.84	84.21 ± 15.16	67.63 ± 9.30	63.10 ± 11.02
Midgut	VH	237.08 ± 37.63	290.41 ± 77.56	301.56 ± 53.90	284.74 ± 52.54	228.90 ± 65.59
	VW	100.44 ± 20.03 ^ab^	104.58 ± 8.44 ^ab^	118.66 ± 34.38 ^a^	99.72 ± 9.22 ^ab^	85.59 ± 10.46 ^b^
	MT	64.23 ± 20.29	65.15 ± 13.34	82.70 ± 24.30	69.77 ± 12.40	64.73 ± 9.57
Hindgut	VH	248.81 ± 40.82	256.85 ± 75.81	295.05 ± 21.94	260.94 ± 52.23	237.35 ± 45.47
	VW	110.14 ± 18.89 ^ab^	112.92 ± 26.22 ^a^	117.64 ± 9.04 ^a^	116.44 ± 18.54 ^a^	86.63 ± 18.69 ^b^
	MT	40.72 ± 9.14 ^b^	54.11 ± 11.33 ^ab^	64.97 ± 17.21 ^a^	58.72 ± 9.68 ^a^	55.31 ± 18.39 ^ab^

Note: Values are presented as mean ± SD (*n* = 3 tanks per treatment). For each tank, measurements were obtained from five randomly sampled fish, and the average value per tank was used for statistical analysis. One-way analysis of variance was used to test the data for significance. In the same row, values with no letter or the same letter superscripts mean no significant difference (*p* > 0.05), while those with different small letter superscripts mean significant difference (*p* < 0.05).

## Data Availability

Data will be made available on request.
